# The human gastric pathogen *Helicobacter pylori *has a potential acetone carboxylase that enhances its ability to colonize mice

**DOI:** 10.1186/1471-2180-8-14

**Published:** 2008-01-23

**Authors:** Priyanka Brahmachary, Ge Wang, Stéphane L Benoit, Michael V Weinberg, Robert J Maier, Timothy R Hoover

**Affiliations:** 1 Department of Microbiology, University of Georgia, Athens, Georgia 30602, USA; 2 Department of Medicine, Division of Diabetes, Endocrinology and Metabolism, Vanderbilt University School of Medicine, Nashville, Tennessee 37232, USA; 3 Merial Ltd., 115 Transtech Drive, Athens, Georgia 30601, USA

## Abstract

**Background:**

*Helicobacter pylori *colonizes the human stomach and is the etiological agent of peptic ulcer disease. All three *H. pylori *strains that have been sequenced to date contain a potential operon whose products share homology with the subunits of acetone carboxylase (encoded by *acxABC*) from *Xanthobacter autotrophicus *strain Py2 and *Rhodobacter capsulatus *strain B10. Acetone carboxylase catalyzes the conversion of acetone to acetoacetate. Genes upstream of the putative *acxABC *operon encode enzymes that convert acetoacetate to acetoacetyl-CoA, which is metabolized further to generate two molecules of acetyl-CoA.

**Results:**

To determine if the *H. pylori acxABC *operon has a role in host colonization the *acxB *homolog in the mouse-adapted *H. pylori *SS1 strain was inactivated with a chloramphenicol-resistance (*cat*) cassette. In mouse colonization studies the numbers of *H. pylori *recovered from mice inoculated with the *acxB:cat *mutant were generally one to two orders of magnitude lower than those recovered from mice inoculated with the parental strain. A statistical analysis of the data using a Wilcoxin Rank test indicated the differences in the numbers of *H. pylori *isolated from mice inoculated with the two strains were significant at the 99% confidence level. Levels of acetone associated with gastric tissue removed from uninfected mice were measured and found to range from 10–110 μmols per gram wet weight tissue.

**Conclusion:**

The colonization defect of the *acxB:cat *mutant suggests a role for the *acxABC *operon in survival of the bacterium in the stomach. Products of the *H. pylori acxABC *operon may function primarily in acetone utilization or may catalyze a related reaction that is important for survival or growth in the host. *H. pylori *encounters significant levels of acetone in the stomach which it could use as a potential electron donor for microaerobic respiration.

## Background

*Helicobacter pylori *is a microaerophilic, gram-negative bacterium that is a significant pathogen of the human gastric mucosa [1,2]. Colonization of the gastric mucosa by *H. pylori *leads to chronic inflammation that can progress to a variety of diseases, including chronic gastritis, peptic ulcer, gastric cancer and mucosal-associated lymphoma [3-5]. In the absence of antimicrobial therapy, the host is likely to suffer a lifetime *H. pylori *infection of the gastric mucosa.

The ability of *H. pylori *to persist in the human stomach for extended periods indicates that it is well adapted to acquire the nutrients it needs for growth in this unique niche. For example, the mucous layer of the mouse stomach contains significant amounts of molecular hydrogen (17–93 μM) originating from metabolic activity of microbial flora in the large intestine [6]. *H. pylori *is capable of utilizing this molecular hydrogen as an electron donor for microaerobic respiration and a functional hydrogenase is required for successful colonization of mice by *H. pylori *[6,7]. Unlike many hydrogen-oxidizing bacteria, however, *H. pylori *is not capable of autotrophic CO_2 _fixation.

Several studies have examined the ability of *H. pylori *to utilize various carbon sources. *H. pylori *has a limited ability to acquire and metabolize sugars, an observation that is consistent with the analysis of the genomic sequences of *H. pylori *strains 22695 and J99 [8]. Glucose is the only carbohydrate that *H. pylori *is capable of utilizing which it does via the Entner-Doudoroff pathway [9,10]. Amino acids also serve as carbon sources for *H. pylori *and are utilized preferentially by *H. pylori *in growth media containing a mixture of glucose and amino acids [9,11]. Pyruvate, a key intermediate in central metabolism, appears to be generated primarily from lactate, alanine and serine, rather than glucose in *H. pylori *[12,13]. Pyruvate is converted to acetyl-CoA by pyruvate:flavodoxin oxidoreductase in *H. pylori*, which can then feed into the tricarboxylic acid (TCA) cycle[14]. In addition, alanine, lactate, acetate, formate and succinate can be produced by *H. pylori *cells incubated aerobically [12]. The production of acetate and formate as metabolic products suggests the existence of a mixed-acid fermentation pathway in *H. pylori*, although oxygen is essential for growth of the bacterium [12].

Analysis of the genome sequences of *H. pylori *strains 26695, J99 and HPAG1 revealed a potential operon of three genes (designated as HP0695, HP0696 and HP0697 in *H. pylori *26695) the products of which shared 50–63% amino acid identity with the β, α, and γ subunits of acetone carboxylase from *Xanthobacter autotrophicus *strain Py2 and *Rhodobacter capsulatus *strain B10 [15]. Homologs of acetone carboxylase are found in a number of bacteria, but the *X. autotrophicus *and *R. capsulatus *enzymes are the best characterized. Acetone carboxylase catalyzes the ATP-dependent carboxylation of acetone to acetoacetate and is required for growth of *X. autotrophicus *and *R. capsulatus *with acetone as the sole carbon source and electron donor for respiration [15-17]. *X. autotrophicus *acetone carboxylase has a high affinity for acetone (Km = 8 μM) but the turnover rate of the enzyme is very slow (~45 per min) [15]. To compensate for the low turnover rate of acetone carboxylase, *X. autotrophicus *produces high amounts of the enzyme (17–25% of total soluble protein) when grown on acetone [15]

Genes located near the *H. pylori *HP0695-HP0696-HP0697 operon encode enzymes shown to convert acetoacetate to acetoacetyl-CoA, which is metabolized further to acetyl-CoA [8,18]. Acetone, acetoacetate and 3-β-hydroxybutyrate are ketone bodies produced by mammalian perivenous hepatocytes during fatty acid degradation and are used as electron donors for respiration when carbohydrates are not readily available [19]. Just as ketone bodies are important energy sources for humans when carbohydrates are not available, these compounds may serve as respiratory electron donors for *H. pylori *colonizing the gastric mucosa. To determine if the HP0695-HP0696-HP0697 operon has a role in host colonization we inactivated HP0696 in *H. pylori *strain SS1. The HP0696 mutant was compromised in its ability to colonize mice suggesting that acetone carboxylation or a related enzymatic activity catalyzed by products of the HP0695-HP0696-HP0697 operon is a significant contributing factor to host colonization.

## Results

### *H. pylori *contains a set of genes predicted to be involved in acetone metabolism

**Figure 1:**
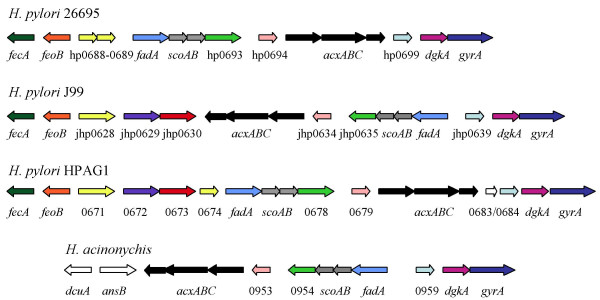
**Organization of the genes involved in acetone metabolism in *H. pylori *and *H. acinonychus *strains**. Gene designations are indicated below each arrow (not drawn to scale). Open reading frames that were not given a gene designation in the annotated genome sequences are indicated with either an hp designation (for *H. pylori *26695), a jhp designation (for *H. pylori *J99), or only the open reading frame number (for *H. pylori *HPAG1 and *A. acinonychis *strain Sheeba). Orthologous genes in the four strains are the same color. The genes jhp0628 in *H. pylori *J99 and 0671 in *H. pylori *HPAG1 correspond to a fusion of hp0688 and hp0689 from *H. pylori *26695. *H. pylori *J99 and HPAG1 have two genes within this region, jhp0629 (HPAG1_0672) and jhp0630 (HPAG1_0672), that encode a type II DNA methyltransferase and a type II restriction enzyme, respectively, and are not found in *H. pylori *26695. Functions of the products of the genes within the acetone metabolism cluster are described in the text. The proposed functions of the products of the surrounding genes are: *fecA*, iron(III) dicitrate transport protein; *feoB*, iron(II) transport protein; *dgkA*, diacylglycerol kinase; *gyrA*, subunit A of DNA gyrase; *dcuA*, anaerobic C_4_-dicarboxylate transporter; and *ansB*, asparaginase II.

**Figure 2:**
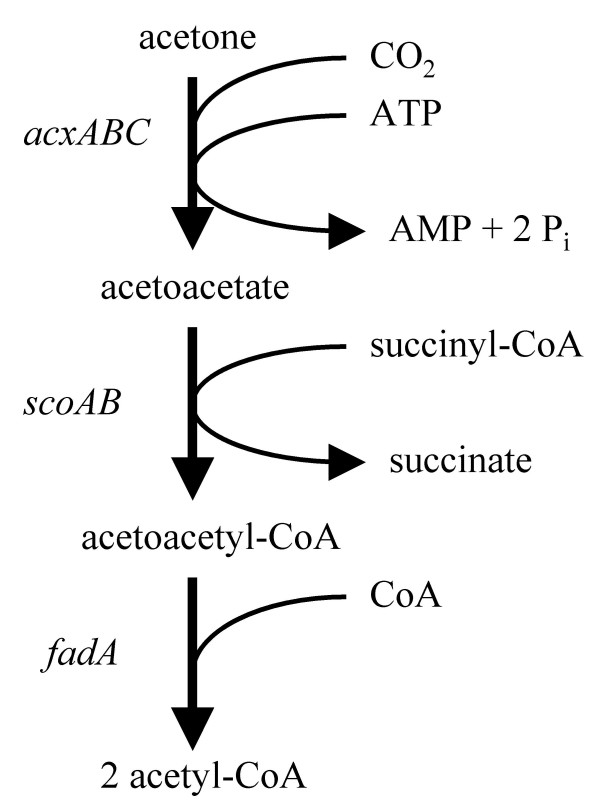
**Proposed pathway for acetone utilization in *H. pylori***. The proposed pathway for the conversion of acetone to acetyl-CoA in *H. pylori *and *H. acinonychis *is shown. Reactions and genes encoding the enzymes responsible for catalyzing each reaction are indicated.

The three *H. pylori *strains whose genomes have been sequenced contain a cluster of eight conserved genes within a ~10 kb DNA sequence, six of which encode enzymes predicted to metabolize acetone and acetoacetate to acetyl-CoA (Figs. 1 and 2). *Helicobacter acinonychis*, a closely related species that infects large felines, also possesses this gene cluster. As indicated above, three of these genes share homology with *acxABC*, although the first two genes in the operon are indicated as genes encoding hydantoin utilization protein A and methylhydantoinase, respectively, in the annotated *H. pylori *genomes. Hydantoinases catalyze the hydrolysis of 5-membered rings via hydrolysis of an internal imide bond and often have fairly broad substrate specificity. Of proteins in the database whose biochemical functions have been demonstrated, BLAST analysis revealed that the predicted products of the *H. pylori *genes most closely match those of the *Xanthobacter *sp. Py2 *acxABC *operon (59–68% amino acid identity over the entire lengths of the three predicted subunits). For the most recently sequenced *H. pylori *and *H. acinonychis *genomes the last gene of the operon is annotated as *acxC *[20,21]. Thus, the *H. pylori *HP0695-HP0696-HP0697 operon probably encodes acetone carboxylase rather than hydantoinase, and we hence refer to this operon as *acxABC*.

Two other genes within the gene cluster, *scoA *(HP0691) and *scoB *(HP0692), encode succinyl CoA:acetoacetate CoA-transferase (SCOT), which catalyzes the conversion of acetoacetate plus succinyl-CoA to acetoacetyl-CoA plus succinate [18]. Acetoacetyl-CoA produced by SCOT is metabolized further by acetoacetyl-CoA thiolase, which is encoded by *fadA *(HP0690; gene is annotated as *thl *in *H. pylori *J99 and *atoB *in *H. pylori *HPAG1 and *H. acinonychis*), to generate two molecules of acetyl-CoA from acetoacetyl-CoA plus coenzyme A (CoA) [8]. The two remaining genes within this cluster, HP0693 and HP0694, are predicted to encode a short chain fatty acid permease and an outer membrane protein, respectively, and may function in transport of acetoacetate.

The eight genes within this putative acetone metabolism cluster are arranged the same relative to each other in the three *H. pylori *strains, but the cluster is oriented in either of the two potential directions (Fig. 1) indicating the occurrence of a DNA inversion in this region during the evolution of *H. pylori*. Alignments of DNA sequences from the three strains narrowed the site of inversion to ~40 bp downstream of the *acxC *homolog and ~160 bp upstream of the start codon for *fadA *(data not shown). No large direct or inverted repeats are found near these regions that might have been involved in inversion, and so we cannot speculate as to the mechanism for this inversion. For *H. pylori *strains J99 and HPAG1, but not 26695, there are a predicted type II DNA methyltransferase (jhp0629 and HPAG1_0673) and a type II restriction enzyme (jhp0630 and HPAG1_0672) adjacent to the putative acetone metabolism gene cluster.

*H. acinonychis *possesses the putative acetone metabolism gene cluster and the genes within the cluster are in the same orientation as those in *H. pylori *J99. In *H. acinonychis *these genes are adjacent to *dgkA *and *gyrA *as they are in *H. pylori*, but are flanked on the opposite end by *dcuA *and *ansB*. The *fecA *and *feoB *genes, which are located near the acetone metabolism gene cluster in the *H. pylori *strains, are ~69 kb from this gene cluster in *H. acinonychis*. Thus, the relative arrangement of the genes within the acetone metabolism cluster has remained remarkably conserved during the evolution of *H. pylori *and *H. acinonychis *despite the fact that the adjacent region in the genomes of these two species has undergone extensive rearrangements.

### The *H. pylori acxB:cat *mutant is deficient in its ability to colonize mice

The *acxB *gene in *H. pylori *SS1, which is a mouse-adapted strain, was disrupted with a chloramphenicol resistance (*cat*) cassette. Cultures of wild-type *H. pylori *SS1 and the *acxB:cat *mutant were grown in Mueller-Hinton broth supplemented with horse serum or a previously described defined medium [22]. Varying amounts of acetone ranging from 1.3 mM to 26 mM were included in the growth media to determine if acetone affected growth of either strain. Cell growth was monitored by viable cell counts as well as optical densities of cultures at various times. Including acetone in either growth media had no effect on the growth rate or final cell yield of *H. pylori *SS1 or *acxB:cat *mutant (data not shown). The failure of acetone to stimulate growth of *H. pylori *SS1 under the conditions tested is not unexpected since the growth media for *H. pylori *is very nutrient rich. These findings also suggest that *acxABC *is not required for acetone detoxification.

**Figure 3:**
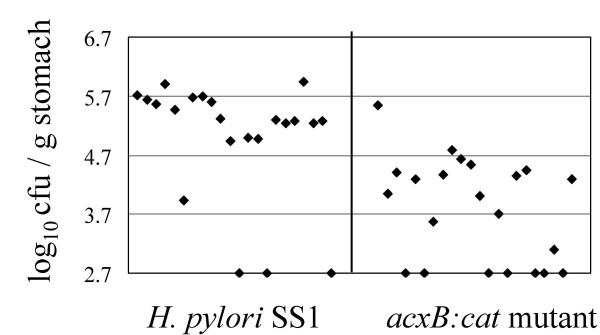
**Mouse colonization assay of *H. pylori *SS1 and an isogenic *acxB:cat *mutant strain**. Data are presented as a scatter plot of colony forming units per gram of stomach as determined by plate counts. Each spot represents the cfu count from one mouse, expressed as the value of log_10 _(cfu/g stomach) in the Y-axis. The base line [log_10 _(cfu/g stomach) = 2.7] is the detection limit of the assay, which represents the count below 500 cfu/g stomach.

The ability of the *acxB:cat *mutant to colonize mice was compared with that of the parental *H. pylori *SS1 strain in two separate trials. In each trial, eleven mice were inoculated with the wild-type strain and eleven were inoculated with the *acxB:cat *mutant. Three weeks post-inoculation of the mice with the *H. pylori *strains, the mice were sacrificed and the numbers of *H. pylori *in the stomachs of the animals were determined. For the mice that had been inoculated with the wild-type strain, most of the animals (19/22 animals) had *H. pylori *counts that were well above the detection limit, which was 500 cfu per gram stomach (Fig. 3). The number of *H. pylori *in samples that were above the detection limit ranged from 10^4 ^– 10^6 ^cfu per gram stomach. Most of the mice inoculated with the *acxB:cat *mutant also had measurable levels of *H. pylori *(15/22 animals), but the numbers of *H. pylori *associated with these mice were generally one to two orders of magnitude lower than those in mice that had been inoculated with the wild-type strain. A statistical analysis of the data using a Wilcoxin Rank test verified that the differences in the numbers of *H. pylori *isolated from mice inoculated with the two strains were significant at the 99% confidence level, indicating that acetone carboxylase enhanced the ability of *H. pylori *SS1 to colonize the mouse stomach. Since we were unable to clone the *acxABC *operon we could not verify by complementation that the *acxB *mutation was responsible for the defect in colonization. However, it is unlikely that the colonization phenotype of the *acxB *mutant was due to polar effects since there are no additional genes in the *acxABC *operon in any of the three *H. pylori *strains whose genomes have been sequenced to date (Fig. 1). Moreover, the colonization defect is not likely due to attenuation or a secondary mutation since we constructed the *acxB *mutant in a fresh isolate of strain SS1 recovered from an infected mouse.

### Acetone levels in the mouse stomach

**Figure 4:**
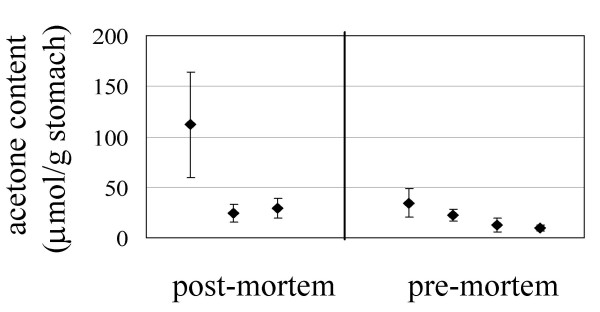
**Acetone levels associated with mouse gastric tissue**. Stomach acetone levels were determined for three mice after sacrificing the animals and immediately removing their stomachs (post-mortem), and for four mice that were anesthetized after which their stomachs were removed (pre-mortem). Excised mouse stomachs were placed immediately in sealed vials that were then placed on ice for at least 30 min to allow acetone associated with the gastric tissue to equilibrate with the gas phase. Acetone levels in the gas phases of the vials were measured by gas chromatography and estimated from standard curves generated for each vial. Each value represents an average of at least three measurements and error bars indicate the standard deviations for each sample.

Since the data from the mouse colonization assays suggested that ability to utilize acetone by *H. pylori *was important for effective host colonization, we wished to determine if *H. pylori *encountered significant acetone levels in the mouse stomach. Although acetone levels have been reported for various bodily fluids, we were unaware of any reports of acetone levels associated with gastric juice or tissue. Therefore, we measured acetone levels associated with mouse gastric tissue after rapidly removing the stomachs from mice and immediately placing the stomachs in sealed serum vials. Since we wished to estimate acetone levels that *H. pylori *could encounter during persistent infection, the animals used for this study were maintained on a regular feeding schedule and were sacrificed in the morning prior to receiving their normal daily food allotment. The sealed serum vials containing the mice stomachs were incubated on ice to allow the acetone associated with the gastric tissue to equilibrate with the gas phase in the vials, after which time gas phase samples were analyzed by gas chromatography. This procedure was done initially by sacrificing the animals and removing their stomachs. Similar results, however, were obtained by removing the stomachs from live animals that had been anesthetized. The amount of acetone associated with gastric tissue for each individual mouse varied, ranging from ~10 to 110 μmols acetone per gram wet weight tissue (Fig. 4), with most values (6/7) falling in the range of 10 and 35 μmols acetone per gram wet weight tissue. These data suggest that millimolar amounts of acetone are associated with mouse gastric tissue and could be available as a potential carbon or energy source for *H. pylori*. This level of acetone associated with the mouse gastric tissue was higher than what we expected since levels of serum ketone bodies vary in humans and other mammals generally range from <0.5 mM to a few millimolar [23]. Acetone is produced in mammals by the spontaneous decarboxylation of acetoacetate and this decarboxylation is enhanced at low pH, and so acetone may accumulate in the stomach due to gastric acidity.

## Discussion

We provide evidence that *H. pylori *possess a functional acetone carboxylase as postulated previously by Ensign and co-workers [15]. The *H. pylori acxABC *operon is near the *scoAB *and *fadB *genes which encode the enzymes SCOT and acetoacetyl-CoA thiolase. Thus, this gene cluster encodes a set of enzymes capable of metabolizing acetone to acetyl-CoA. Acetyl-CoA produced from acetone and acetoacetate could feed into the TCA cycle to provide energy for *H. pylori*. As observed by Pflock and co-workers the *acxABC *operon and other genes associated with acetone metabolism are present in *H. acinonychis *[24]. These genes are absent, however, in the other closely related ε-Proteobacteria whose genomes have been sequenced so far, which includes *Helicobacter hepaticus*, *Campylobacter jejuni*, *Thiomicrospira denitrificans*, and *Wolinella succinogenes*. The acquisition and maintenance of the acetone metabolism gene cluster in *H. pylori *and *H. acinonychis *may be related to the fact that, unlike these other related ε-Proteobacteria, they colonize the stomach mucosa. The clustering of these genes and the absence of orthologous genes in other closely related ε-Proteobacteria could indicate possible acquisition of these genes by *H. pylori *and *H. acinonychus *through lateral gene transfer. The G+C content of this cluster of acetone metabolism genes in *H. pylori *is slightly higher than the average for the whole genome, but this appears due to the products of these genes being very rich in glycine (~10% compare to 6% genome average). Moreover, compositional characteristics of these genes, such as dinucleotide relative abundances and codon bias, are not indicative of recent lateral transfer of this region [25] (J. Mrázek, personal communication).

Jungblut and co-workers reported that *H. pylori *AcxC (HP0698) cross-reacted with antibodies from an adenocarcinoma patient, indicating that the *H. pylori acxABC *operon is expressed in the host [26]. Moreover, we show here that *H. pylori *can encounter significant levels of acetone in the mouse stomach and so this compound may serve as an important respiratory electron donor for the bacterium in the host. Consistent with this hypothesis, the *H. pylori acxB *mutant was significantly reduced in its ability to colonize the mouse stomach which we infer results from the inability of the mutant to utilize acetone as an energy source. The inability of acetone to stimulate growth of *H. pylori *SS1 in liquid culture may result from the failure of the growth media used to mimic growth conditions encountered by the bacterium when colonizing the gastric mucosa. An alternative hypothesis for the colonization defect of the *acxB *mutant is that acetone carboxylase is needed for detoxification of acetone. However, our failure to observe any inhibition in growth of the *acxB *mutant by the addition of acetone in the culture medium argues against this latter hypothesis. Another possibility is that the products of the *acxABC *operon catalyze an unknown reaction that is important for survival or cell growth of *H. pylori *in the gastric mucosa. Further biochemical characterization of the products of the *H. pylori acxABC *operon should help to distinguish between these possibililties.

A recent transcriptome analysis of *H. pylori *26695 using a whole genome microarray suggested that the response regulator HP1021 strongly activated transcription of *acxABC *and *scoAB*, and activated transcription of *fadA *and hp0693 to a lesser extent [24]. The authors of this study showed that HP1021 binds the promoter regulatory region of *acxABC*, suggesting that this response regulator directly mediates its effects on transcription of *acxABC *and that the genes within the acetone metabolism cluster are part of a regulon controlled by HP1021. Pflock and colleagues identified 79 genes in *H. pylori *26695 whose expression was altered in the HP1021 mutant – 51 genes were transcribed at lower levels in the mutant while 28 genes were expressed at higher levels [24]. HP1021 differs from most other response regulators in that it lacks the highly conserved phosphate-accepting aspartate residue, and a cognate histidine kinase for HP1021 has not been identified. There are conflicting reports on the transcription of HP1021 in response to acidic pH, as well as on the transcription of *H. pylori acxABC *in response to lower pH [27-29]. These differences may be due to the way in which the bacteria were cultured. Although these reports conflict with regard to transcription of HP1021 in response to acidic pH, results from both studies indicate that conditions that lead to down-regulation of HP1021 result in increased expression of *acxABC*. This seems counterintuitive given the apparent role of HP1021 in activating transcription of *acxABC*.

The only other bacterium for which regulation of the *acxABC *operon has been examined is *X. autotrophicus*. Transcriptional control of *acxABC *in *X. autotrophicus *differs from that in *H. pylori*. *X. autotrophicus *lacks a homolog of HP1021, but rather regulates transcription of *acxABC *via σ^54 ^(RpoN) and the σ^54^-dependent activator AcxR [15]. Although *H. pylori *possesses σ^54^, the *acxABC *operon is not part of the *H. pylori *RpoN regulon [30].

Despite our observation that disruption of *acxB *adversely affects colonization of mice by *H. pylori *SS1, recent whole genome microarray studies with fifty-six globally representative strains of *H. pylori *and four *H. acinonychis *strains indicated that the *acxABC *genes are not present in all *H. pylori *strains [31]. It would be interesting to determine if the isolates lacking *acxABC *are less competitive in colonizing their natural hosts than strains that possess these genes. Alternatively, strains lacking *acxABC *may have adaptations that compensate for the lack of acetone carboxylase activity. Results from the microarray studies by Gressmann and colleagues indicated that *scoAB*, *fadA*, HP0693 and HP0694 were present in all the *H. pylori *and *H. acinonychis *strains examined [31]. Thus, the selective pressure of maintaining the ability to utilize acetoacetate as a potential electron donor in *H. pylori *and *H. acinonychis *appears to be greater than that for acetone metabolism.

## Conclusion

The *H. pylori acxABC *operon likely encodes acetone carboxylase that catalyzes the conversion of acetone to acetoacetate and is intimately associated with genes whose products are predicted to catalyze the sequential conversion of acetoacetate to acetyl-CoA. Inspection of genomes of other closely related ε-Proteobacteria suggests that genes involved in acetone metabolism are only present in bacteria within this subphylum that colonize the gastric mucosa. The *acxABC *operon was not essential for mouse colonization by *H. pylori *SS1, but it did appear to enhance colonization. Further characterization of the putative *H. pylori *acetone carboxylase and the products of the other genes within the acetone metabolism gene cluster should provide insight into how ketone bodies from the host contribute to the metabolic economies of *H. pylori *and *H. acinonychis *and how these compounds impact the ability of these bacteria to colonize their hosts.

## Methods

### Bacterial strains and media

Plasmid construction and cloning was done in *E. coli *strain DH5α which was cultured in Luria-Bertani medium at 37°C. *H. pylori *strain 26695 was used as the template for polymerase chain reaction (PCR). *H. pylori *SS1 was used as the wild-type strain for all experiments and was cultured on either blood agar or tryptic soy agar supplemented with 5% horse serum (TSA-serum) at 37°C under an atmosphere of 4% O_2_, 5% CO_2 _and 91% N_2_. When cultured in liquid medium, *H. pylori *cultures were grown in Mueller-Hinton broth supplemented with 5% horse serum and 30 μg/ml bacitracin or the defined growth medium described by Bruggrabber and co-workers [22]. Cultures (10–15 ml grown medium) were grown in 150-ml serum vials sealed with 20 mm Teflon/silicone discs and aluminum caps and under an atmosphere of 4% O_2_, 5% CO_2_, 10% H_2_, 81% N_2_. Unless indicated otherwise, when antibiotics were included in the medium they were added to the following concentrations: 100 μg/ml ampicillin, 30 μg/ml chloramphenicol, 200 μg/ml bacitracin 10 μg/ml vancomycin, and 10 μg/ml amphotericin B.

### Inactivation of *acxB *(HP0696) in *H. pylori *SS1

A 2.3-kb DNA fragment that carried *acxB *was amplified by PCR from *H. pylori *strain 22695 and cloned into pGEM-T (Promega). A *cat *cassette was introduced into this plasmid at an *Eco*47III site located approximately in the middle of the cloned *acxB*. The resulting plasmid was used as a suicide vector for inactivating the chromosomal copy of *acxB *in *H. pylori *SS1. The suicide vector was introduced into *H. pylori *strains ATCC 43504 and SS1, a strain that can colonize mice. Because repeated passage of *H. pylori *strain SS1 on medium has been reported to result in loss of infectivity in mice, the *acxB *mutant was constructed in a fresh isolate of strain SS1 recovered from an infected mouse. The number of passages of *acxB *mutant in the SS1 strain was limited and recorded, and the mutant was stored frozen at -80°C. The parental SS1 strain was maintained and stored frozen in an identical manner. We confirmed by PCR that the chromosomal copy of *acxB *was disrupted by allelic exchange with the plasmid-borne copy of the gene using a set of primers that flanked the site of disruption.

### Growth curves for *H. pylori *strains

*H. pylori *cells from TSA-serum plates on which the strains had been streaked on the previous day were suspended in phosphate-buffered saline (PBS) and used to inoculate liquid medium at an OD_600 _of 0.03. Where indicated, acetone was added aseptically to the medium. Samples were taken at various times and cell densities were measured by light scattering at OD_600_. Alternatively, viable cell counts were determined following serial dilution of the samples and plating on TSA-serum. Following 4 to 5 days incubation, the numbers of colony forming units (cfu) were determined for the plates.

### Mouse colonization

Mouse colonization assays were performed essentially as described earlier [32]. These procedures complied with the relevant federal guidelines and institutional policies for the care and handling of laboratory animals. Briefly, *H. pylori *cells were harvested after 48 h of growth on blood agar plates and suspended in PBS to an OD_600 _of 1.7. Headspace in the tube was sparged with argon to minimize oxygen exposure. These suspensions were administered to C57BL/6J mice via oral gavage. The mice were inoculated with *H. pylori *two times (two days apart) with a dose of 1.5 × 10^8 ^bacterial cells/mouse. The inoculum dose was determined from reproducible standard curves of OD_600 _versus viable cell number from plate counts. Three weeks after the first injection, the mice were sacrificed and the stomachs were removed, weighed, and homogenized in argon-sparged PBS. Homogenates were diluted serially and the dilutions were plated on blood agar supplemented with vancomycin, amphotericin B and bacitracin (100 μg/ml). The plates were incubated for 5–7 days in an incubator containing 2% O_2_, 5% CO_2 _and the balance N_2_, after which the plates were examined for *H. pylori *colonies.

### Measuring acetone levels in mouse stomachs

Stomachs were surgically removed from pre-mortem and post-mortem C57BL/6J mice (Jackson Labs) and placed immediately in small glass vials which were half-filled with glass beads to minimize the head space. The vials were sealed with 20 mm Teflon/silicone discs and aluminum caps and place on ice. Samples from the head space were analyzed with a gas chromatograph equipped with a flame ionization detector (Model GC-8A, Shimadzu). Samples were removed from the head space with 1 ml VICI Pressure-Lok Precision analytical syringe (Precision Sampling) and injected into a DB624 column (30 m × 0.53 mm, 3 μm mesh). The injector and the detector temperatures were 250°C and air was used as the carrier at 0.4 kg/cm^2^. Retention times and peak areas were recorded with a Chromatopac (Model CR601, Shimadzu), and the retention time for acetone under these conditions was ~0.61 min. For each sample a separate acetone standard curve was prepared as follows. The mouse stomach was removed from the vial and washed extensively in ice cold PBS. The stomach was placed back in the vial and 100 μl of a known amount of acetone was applied to the tissue. The vial was resealed and placed on ice, and samples from the head space were analyzed by gas chromatography. The stomach was removed from the vial and the procedure was repeated with a different known amount of acetone. All procedures involving mice complied with the relevant federal guidelines and institutional policies for the care and handling of laboratory animals.

## List of abbreviations

cfu – colony forming units

CoA – coenzyme A

PBS – phosphate buffered saline

SCOT – succinyl CoA: acetoacetate CoA-transferase

TCA – tricarboxylic acid cycle

TSA – trypic soy agar

## Authors' contributions

PB constructed the acxB:cat mutant and measured acetone levels in the mouse gastric tissue. Mouse colonization assays were done by GW and RJM. Growth analysis experiments were done by SB and MVW. TRH conceived of the study, participated in the design and coordination of experiments, and helped draft the manuscript. All authors read and approved the final manuscript.
